# Toward tailored interventions in plantain (*Musa paradisiaca* L.) industry: Insights from heterogeneity and constraints to plantain-based cropping systems in South-Benin

**DOI:** 10.1016/j.ssaho.2024.100895

**Published:** 2024

**Authors:** Adikath Abiola, Ygué P. Adégbola, Martine Zandjanakou-Tachin, Géraud F. Crinot, Gauthier Biaou

**Affiliations:** a National University of Agriculture (UNA), PO BOX 043, Ketou, Benin; b National Institute of Agricultural Research of Benin (NIARB), PO BOX 884, Benin; c International Center for Research and Training in Social Sciences (CIRFOSS), Abomey-Calavi, Benin

## Abstract

**Figure:**
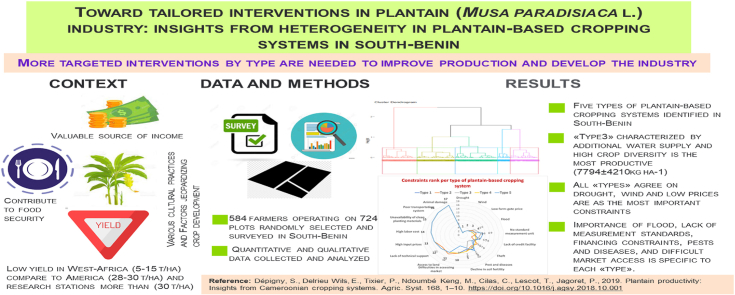

## 1 Introduction

In Africa as well as worldwide, agriculture plays three core functions: reducing poverty, fostering economic growth, and ensuring food security (Adégbola et al., 2017). As actual fact, agricultural development is estimated to be 3.2 times more effective in terms of poverty alleviation than any other sector, thereby, improving households' food security and nutrition (Maï et al., 2020). Among the wide range of crops provided in this sector, plantain is an important source of food and income for millions of people worldwide and particularly in Sub-Saharan Africa (Sodedji et al., 2016). In Benin, the crop has long held a marginal place and was considered as a secondary crop (Lokossou & Achigan, 2000). It was then produced around huts as a backyard crop (Lokossou & Achigan, 2000). Recently, different actors in agriculture show their interest in the crop, owing to its high market value and his high-income stability potential (Hedegla, 2022). From a production, mostly self-consumed, plantain production is mostly targeting market. Also, it got more importance between farmers crop choices (Dassou et al., 2021). Consequently, from the backyard garden, plantain production is gradually shifting to more diversified production practices and cropping systems. This is triggered by the high demand of the raw plantain and processed products due to the growth of the food processing sector (Hedegla, 2022; Ojokojo, 2016). The crop entails a considerable economic opportunity for rural households and other actors of the food chain. Despite the high economic potential of the crop, its production is mainly extensive and low compared to other african producing countries (Chabi et al., 2018; Faï et al., 2018). With a production estimated to 20138.06 tons, in 2020, Benin is a small producer compared to the sub-region countries like Nigeria, Ghana, Ivory-Coast and Cameroon with respectively, 3.07, 4.66; 1.88 and 4.52 million tons (FAOSTAT, 2020). Nevertheless, Benin shares some agroecological similarities with at least Nigeria (Houngbo et al., 2015). To reinforce plantain production, the government established a new agricultural policy by implementing the National Program of Plantations and high-value Crops Development (PNDPC) (PRB, 2021). Hedegla (2022) and Lokossou and Achigan (2000) signposted three basic elements to work out on plantain crop chain to achieve the PNDPC objectives: organizing farmers into cooperatives, marketing efficiency, support and training to farmers. The latter was the utmost recommendation in Hedegla (2022) as the author revealed that the poor knowledge of plant botany and physiology, production techniques, and pest management, exacerbated by drought and seasonal winds are limiting factors to plantain production. These recommendations are supported by a one-size-fits-all approach, implying a general and equal weights attributed to each constraint. Also, the lack of reliable information (eg. typology, structuring, functioning, production, access to agricultural services) in agriculture in Benin, and particularly on banana production, is considered as a constraint to the development of the agricultural sector (Sossou et al., 2021). To better guide decision-makers and foster the design of sound agricultural policies, it is important to assess farms diversity through the typology of plantain-based cropping systems. In actual fact, there is a significant heterogeneity within the farm system that can mislead the interpretation of factors affecting farm performance and impedes development objectives (Adegbola et al., 2022; Ferraton & Touzard, 2009). Differentiation through typology is thus crucial to assess farmers' opportunities and constraints (Dé et al., 2019; Hammond et al., 2020a; Sero et al., 2020). It controls the heterogeneity within the farm by forming homogeneous « types » that can be compared to others (Adegbola et al., 2022; Loyola et al., 2018). Farmers grouped into homogenous types are showed to likely respond in a similar manner to a policy intervention (Adégbola et al., 2017; Adegbola et al., 2022). In addition, typology allows a better understanding of the farmers’ realities and the recommendation of appropriate solutions. Pacini et al. (2014) showed that typologies can help design a range of solutions that are relevant and adjusted to the needs and means of different types of farms. Further, by taking into account within-region heterogeneity, typologies allow to understand how appropriate interventions can be disseminated at a large scale (Alvarez et al., 2014, 2018). Moreover, «peasant economy» and «subjective equilibrium » theories support the importance of this differentiation to determine targeted intervention mechanisms for a sustainable crop chain (Ferraton & Touzard, 2009; Hammond et al., 2020b; Chayanov, 1966; Tanaka et al., 1951). We therefore hypothesized that, accounting for the diversity in plantain-based cropping systems will lead to the identification of constraints which importance will differ from a type to another.

Literature review showed that the existing literature poorly addressed this thematic in banana and plantain production and in Benin (Abiola et al., 2020; Akerele et al., 2019; Blazy et al., 2009; Chabi et al., 2018; Dé et al., 2019; Faï et al., 2018; Patel et al., 2020). As matter of fact, previous studies used the statistical approach (Ahouangninou et al., 2021; Bellamy, 2013; Loyola et al., 2018), while analyzing in a general way the limiting factors in banana and plantain production (Akinyemi et al., 2013; Baruwa et al., 2015; Dassou et al., 2021; Muthee et al., 2019; Nandwani, 2010). Most of studies carried out in Benin are limited to a relatively small territorial scale and these studies have not been carried out at the scale of Agricultural Development Hubs (ADH) which is the new framework for the implementation of agricultural development policies, programs and projects (MAEP et al., 2017). The present study is carried out to fill this gap and to identify different plantain-based cropping systems taking into account ADH, the problems they face, and the policy implications specific to each group.

This study innovates in the typology construction by using a robust econometric method. Calinski and Harabasz pseudo-F-index was used to identify the optimal number of clusters. The bootstrapping approach was applied and is considered as an improvement to ensure the stability of the clustering results (Mingoti & Felix, 2009; Álvarez-Ló et al., 2008). The combination of these techniques allowed us to objectively determine the optimal number of robust plantain-based cropping systems (Mingoti & Felix, 2009; Yu et al., 2019). Having the capacity to use both quantitative and qualitative data, this econometric method is able to treat several discriminating factors at the same time, and makes it possible to analyze the complexity of cropping systems, and above all to determine the most representative of each system. This approach has been used in researchs in Social Sciences and operational management such asMachado-Bouroullec et al. (2016), FAO (2019), FAO/CIRAD (2017), Granular (2023), and Pot (2018). Furthermore, by accounting for this diversity in the constraints analysis, this study helped to identify the priorities in intervention for each type. This contribute to the debate on “one-size does not fit all” approach, suggesting tailored interventions (Hammond et al., 2020b; World Bank, 2016). The rest of this article is organized as follows: The next section provides an overview of the methology used in the study. It embodies the study area and sampling, data collection and data analysis. The latter describes step by step the procedure used to construct the typology and the method used to analyze the constraints. The empirical results are presented in the third section and discussed in the fourth section. The article ends with concluding remarks and perspectives.

## 2 Material and methods

### 2.1 Study area and sampling

**Fig. 1:**
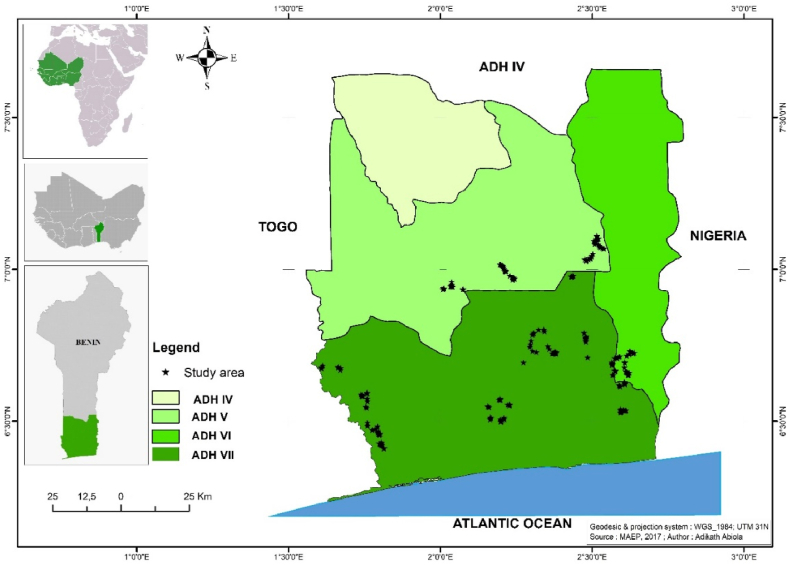
Map of the plantain production area studied.

Located between the Equator and the Tropic of Cancer, Benin is a west african country covering 112,763 km^2^ of area. The country is located between the latitudes of 6°30′ and 12°30′ N, and the longitudes of 1° and 30°40′ E (Adegbola et al., 2022). Benin is divided into seven ADHs, of which, three, namely the ADHs 5, 6 and 7 have been selected to conduct a survey (MAEP, 2017). These areas are suitable for banana and plantain cultivation (Dassou et al., 2021) and are part of the “banana belt” identified in the West Africa (Cauthen et al., 2013). Moreso, banana and plantain are highly produced and marketed in the area (Lokossou & Achigan, 2000). Similarly to previous studies, five departments were selected within the ADHs visited: Mono, Atlantique, Zou, Ouémé and Plateau (Chabi et al., 2018; Dassou et al., 2021; Faï et al., 2018; Lokossou et al., 2012a). Fig. 1 presents the study area.

Furthermore, the study used the multi-stage random sampling method, from districts to villages. Following previous studies on banana and plantain, 2 districts were selected per department, and 6 villages were selected in each district (Abiola et al., 2020; Chabi et al., 2018; Dassou et al., 2021). To select plantain farmers, available data sets were collected from the Territorial Agency of Agricultural Development (ATDA) and projects on banana and plantain. After combining the data sets and extracting plantain farmers lists, 10 plantain farmers were randomly selected by village. Due to the death of some farmers, the relocation of others and the abandonment of the activity by some, only 584 farmers operating on 724 plots were surveyed instead of 600. The sample size has been determined following Dillman (2007):

(1) $$n=\frac{t_{P}^{2}.P\left(1-P\right).N}{t_{P}^{2}.P\left(1-P\right)+\left(N-1\right).e^{2}}$$

Where n is the sample size; N is the target population size; P is the expected proportion of a response among the population; e is the acceptable margin of error; and $t_{P}$ is the confidence interval.

### 2.2 Data collection

An exploratory survey has been carried out from February 23 to 28, 2021 to collect qualitative data through focus groups of 4–6 people. The data were related to the existing cropping systems, the cultivation practices, the factors limiting plantain production, their causes and consequences. Subsequently, a quantitative survey of plantain farmers was conducted from July 21 to October 4, 2021 to collect relevant quantitative data for typology and characterization as well as constraints. We used a structured interview, with a questionnaire, previously pre-tested, to collect the data. Collected data included information on input quantities, production factors, plot characteristics, water management, agricultural practices, cropping system and constraints to plantain production (Akinyemi et al., 2010, 2013; Azonkpin et al., 2018; Dassou et al., 2021; Dorvil et al., 2023; Lemeilleur et al., 2003; Muthee et al., 2019; Oso et al., 2014; Zongo et al., 2016).

The constraints identified in previous studies and in the exploratory survey were ranked by farmers in the quantitative survey in terms of their importance (Akinyemi et al., 2013; Baruwa et al., 2015; Dassou et al., 2021; Nandwani, 2010; Patel et al., 2020; Schill et al., 2000).

### 2.3 Data analysis

#### 2.3.1 Typology of plantain-based cropping systems

The Mixed Data Factor Analysis (MDFA) is a statistical technique used to analyze datasets that contain both categorical and continuous variables. It allows the exploration of relationships between different types of variables in an unified framework. To determine the optimal number of clusters, the Calinski-Harabasz pseudo-F statistic was employed. This statistic measures the between-cluster dispersion relative to the within-cluster dispersion, providing an indication of the compactness and separation of the clusters. By comparing the pseudo-F values for different numbers of clusters, one can select the number of clusters that maximizes the separation between clusters while minimizing the within-cluster variation. Additionally, the bootstrapping method was used to assess the stability of the identified clusters. Bootstrapping involves repeatedly resampling the data with replacement to create multiple bootstrap samples. By applying MDFA to each bootstrap sample, it becomes possible to examine the consistency and robustness of the clustering solution across different subsamples of the data. This helps to evaluate whether the identified clusters are reliable and not heavily influenced by random variations in the data. Finally, the ANOVA test was used to compare the characteristics of the obtained clusters. ANOVA (Analysis of Variance) is a statistical test that assesses whether there are significant differences between the means of different groups. In this case, the ANOVA test was similarly applied to the categorical and continuous variables separately, comparing the average values across the identified clusters. It helps determining if there are statistically significant differences between the clusters in terms of these variables. Overall, the combination of MDFA, the Calinski-Harabasz pseudo-F statistic, bootstrapping, and ANOVA provides a comprehensive approach for identifying distinct types or clusters within datasets containing both categorical and continuous variables.

All statistical analyses and graphical outputs were obtained using the R software (version 4.1.1). The typology was constructed through the following steps.

##### 2.3.1.1 Selection and standardization of variables

The classification started with the identification and selection of variables that are likely to be relevant to describe plantain-based cropping systems. This process was based on the recent literature on the typology of cropping systems in general and plantain in particular (Ahouangninou et al., 2021; Allagbe et al., 2014; Azonkpin et al., 2018; Dorvil et al., 2023; Fondio et al., 2011; Jouve, 2003, 2006; Lokossou et al., 2012a; Ouikoun et al., 2019; Zongo et al., 2016). A total of 14 variables were selected and are described in Table 1. Since multivariate analysis methods are sensitive to the presence of outliers, the first step was to check it. Therefore, an exploratory data analysis was performed through histograms, boxplots and frequency analysis for the purpose, and also to explore variables distribution as well as strong correlations.

**Table 1:** Description of variables used in the typology and characterization.

Variables	mean (SD)	Description
Quantity of urea	4.40 (25.26)	Continuous (kg/ha)
Quantity of NPK	4.61 (26.16)	Continuous (kg/ha)
Irrigated lowland	–	Dummy, 1 = Yes (N = 60), 0 = No (N = 664)
Flat land	–	Dummy, 1 = Yes (N = 501), 0 = No (N = 223)
Plot irrigation	–	Dummy, 1 = Yes (N = 118), 0 = No (N = 606)
Quantity of hired labor	27.87 (563.98)	Continuous (Man/day)
Quantity of total labor	42.84 (610.87)	Continuous (Man/day)
Quantity of organic fertilizer	103.95 (550.50)	Continuous (kg/ha)
Age of plantations	5.65 (5.04)	Continuous (year)
Area of associated crops	0.23 (0.46)	Continuous (ha)
Sowing method	–	Dummy, 1 = Natural mat expansion (N = 173), 2 = Row seedling (N = 378), 3 = Staggered seedling (N = 173)
Plantation density	–	Dummy, 1 = Low <1200 plants/ha (N = 603), 2 = [1200–1500 plants/ha] (N = 121)
Cropping method	–	Dummy, 1 = Relay cropping (N = 14), 2 = Intercropping (N = 31), 3 = Mixed cropping (N = 391), 4 = Monocropping (N = 288)
Tractor use	–	Dummy, 1 = Yes (N = 45), 0 = No (N = 679)

##### 2.3.1.2 Mixed Data Factor Analysis (MDFA)

Factor analysis is a data reduction technique that reduces a large number of variables into small numbers of uncorrelated factors that have greater explanatory power. The technique encompasses three set of methods, each used, depending on the type of variables. The principal Component analysis (PCA) is used for quantitative (continuous) variables, the Multi-correspondence Analysis (MCA) is used for qualitative (categorical) variables, and the Mixed Data factor Analysis (MDFA) is applied on both quantitative and qualitative variables (Mixed dataset) (Adegbola et al., 2022; Kassa et al., 2017; Pagès, 2004). Since the variables selected in this study include both quantitative and qualitative variables, the Mixed Data Factor Analysis (MDFA) function of the ‘FactoMineR’ package in R 4.1.1. software was used (Lê et al., 2008). After eliminating variables with low variation, 11 variables were used in the MDFA. The factor scores with an eigenvalue greater than one (according to the Kaiser criterion) were used as input data for the cluster analysis to generate a typology. In the final step, each factor was interpreted on the basis of the original variables with a factor loading greater than 0.5.

##### 2.3.1.3 Cluster analysis

Hierarchical clustering is used to determine the number of homogenous groups or « types » based on selected criteria and variables. It is used to group observations and identify trends in the data. Using Ward's method and the Euclidean distance matrix in the hierarchical classification algorithm, the number of homogenous groups or « types » was determined. An advantage of the ward's method is that it is not sensitive to small distortions in the data (Adegbola et al., 2022). The optimal number of clusters can be obtained either with a subjective inspection of the dendrogram or by a statistical test. The study used both. First, the Calinski and Harabasz pseudo-F statistic (Calinski & Harabasz, 1974) was used to select the optimal number of clusters. The number of clusters with the highest Calinski-Harabasz index value is the most likely number of clusters (Adegbola et al., 2022; Mingoti & Felix, 2009). Then, the dendrogram was subjectively inspected to confirm the result.

##### 2.3.1.4 Assessment of the reliability of the clustering results

The non-parametric bootstrapping Jaccard index was used to confirm or deny the choice of the optimal number of types. It helps to confirm the number of types that maximize the stability of the clusters. A wrong choice can lead to bad typological results and a wrong decision (Mingoti & Felix, 2009; Álvarez-Ló et al., 2008). Thus, it is important to ensure that the typology result represents the real structure of the data and not an artefact of the classification algorithm (Adegbola et al., 2022). Thus, the estimate of the Jaccard stability index of each type of plantain-based cropping system in the original classification was calculated, using re-sampling to compare original sample and the bootstrapped subsample solutions. The original clustering's Jaccard stability index estimate for each type of plantain-based cropping system was computed as the mean value of its maximal Jaccard coefficient over all bootstrap iterations (Mucha, 2016). As a general rule of thumbs, types with a Jaccard stability index value less than 0.60 are considered unstable. They are considered very stable if their stability values are at least 0.85 (Adegbola et al., 2022). When analyzing the stability of the typology outcomes, the bootstrap method is more efficient. Moreover, it offers a nonparametric maximum likelihood estimate (MLE) of the clustering instability. It also outperforms all other algorithms at hierarchical cluster analysis (Adegbola et al., 2022; Fang & Wang, 2012; Mucha, 2016). Besides, the Jaccard coefficient is straightforward to interpret and is independent of the number of plantain farms that belong neither to the bootstrapped sample nor to the original sample. One drawback of the method, is the risk of overestimating the stability (Adegbola et al., 2022; Yu et al., 2019).

#### 2.3.2 Analysis of constraints to plantain production

Data from the literature review (Blazy et al., 2009; Chabi et al., 2018; Dassou et al., 2021) and qualitative interviews made it possible to identify the list of constraints which were subsequently ranked by the respondents during quantitative interviews. Subsequently, rank data obtained were analyzed using Kendall's rank correlation method with SPSS Software. Indeed, for k sets of hierarchies (observations) and N hierarchical objects (constraints), the association between these ranks can be determined from Kendall's concordance coefficient W. This coefficient provides the degree of association among these k sets. Therefore, it is used to evaluate the degree of agreement between observations. Considering a matrix k×N with k rows (assigned ranks) and N columns (constraints). The coefficient W is expressed as follows:

(2) $$W=\frac{\sum {\left(R-\bar{R}\right)}^{2}}{k^{2}\left(N^{3}-N\right)/12}$$

With $\bar{R}$ the average rank obtained for all observations per constraints.

Between 0 and 1, W indicates a high degree of agreement when it is close to 1. According to Schmidt-Roy (Schmidt-Roy, 1997), the degree of agreement is high for W = 0.7 and acceptable from W = 0.5. The significance of W can be evaluated by comparing, at 5% or 1% significance level and at degree of freedom df=N−1, the theoretical Chi-square value to the observed Chi-square value, obtained as:

(3) $$x^{2}=k\left(N-1\right)W$$

It also allows one to conclude with considerable confidence that the consensus between the *N* sets is higher than it would have been by chance if their prioritization had been random or independent.

Note that the semi-structured interviews conducted during the exploratory phase yielded transcribed verbatims relative to the constraints, which were subjected to a content analysis using the ATLAS.ti software (version 8). Content analysis is a qualitative research tool that involves the systematic coding and categorization of textual data according to themes or topics (Assarroudi et al., 2018). The content analysis was performed using the following steps.

##### 2.3.2.1 Preparation of data

The verbatims were imported into ATLAS.ti and organized into sources. Each source corresponded to a focus group transcript. The sources were then classified into cases, which represented the different districts where the focus groups were conducted. The cases were assigned attributes such as the zone name, the number of participants, and the date of the interview.

##### 2.3.2.2 Coding of data

The coding process involved assigning labels or codes to segments of text that reflected their meaning or content. The codes were created based on the research questions and the objectives of the study, as well as the emerging themes and patterns from the data. The codes were organized into a hierarchical structure, with parent codes representing broader categories and child codes representing subcategories or specific aspects. The coding was done manually, by selecting relevant text segments and assigning them to existing or new codes. The coding scheme was refined and revised throughout the coding process, by adding, deleting, merging, or splitting codes as needed.

##### 2.3.2.3 Analysis of data

The analysis of data involved exploring the coded data and generating insights and findings. The analysis was guided by the research questions and the objectives of the study, as well as the literature review. The analysis used various tools and techniques available in ATLAS.ti, such as queries, matrices, charts, and maps. Queries are used to search for specific words, phrases, or codes in the data, and to filter or compare the data based on certain criteria. Matrices are used to display the frequency or percentage of codes or cases across different attributes or categories. Charts are used to visualize the distribution or relationship of codes or cases in different formats, such as bar charts, pie charts, or cluster analysis charts. Maps are used to create graphical representations of the data, such as mind maps, concept maps, or project maps. These tools and techniques helped to identify and describe the main constraints to plantain production in each district, as well as their causes and consequences. The identified constraints were thoroughly investigated during the quantitative survey, where each of them were ranked based on their importance for farmer.

## 3 Results

### 3.1 Typology of plantain-based cropping systems

#### 3.1.1 Mixed Data Factor Analysis (MDFA)

Table 2 presents the results of the Mixed Data Factor Analysis. Eight dimensions with an eigenvalue greater than 1 are extracted from the initial 14 variables. The results show that the eight factors explain 72.74% of the total variance. The first factor is correlated with urea quantities (0.50), NPK quantities (0.58), flat land (0.59), irrigated lowland (0.59). This factor represents “*Chemical fertilization and ecology*” and explains 14.87% of the total variance. The second factor relates to crop irrigation, with a correlation of 0.55. The factor “*Irrigation system*”, explains 12.14% of the total variance. The third factor, representing “*Labor intensity*”, is associated with the quantities of total labor and the hired labor, with correlations of 0.64, respectively. This factor represents 11.09% of the total variance. The fourth factor (“*Organic fertilization*”) is associated with the quantities of organic fertilizer and age of plantation, and explains 8.57% of the total variance. The fifth factor, “*Extent of crop association*”, is associated with the area of associated crops (0.58). The sixth factor is associated with sowing method (0.55). The seventh factor, “*Crop diversity*”, is correlated with plantation density and cropping method, with correlations of (0.55) and (0.70) respectively. This factor accounts for 5.99% of the variance explained. Finally, the eighth factor, “*Mechanization*”, is associated with the use of tractors for ploughing and soil preparation. This factor represents 5.80% of the total variance.

**Table 2:** Selected factors with their respective eigenvalues and percentage of variance explained.

Variables	Factors							
	CF	IS	LI	OF	ECA	ST	CD	M
Quantity of urea (kg/ha)	**0,50**	0,19	0,19	0,00	0,20	0,00	0,00	0,00
Quantity of NPK (kg/ha)	**0,58**	0,17	0,20	0,01	0,21	0,00	0,00	0,00
Irrigated lowland (%yes)	**0,59**	0,38	0,01	0,00	0,00	0,01	0,01	0,00
Flat land (%yes)	**0,59**	0,27	0,01	0,00	0,00	0,02	0,00	0,00
Plot irrigation (%yes)	0,31	**0,55**	0,01	0,04	0,03	0,00	0,00	0,00
Quantity of hired **labor** (Man/day)	0,11	0,19	**0,64**	0,01	0,00	0,00	0,00	0,00
Quantity of total **labor** (Man/day)	0,10	0,19	**0,64**	0,02	0,00	0,00	0,00	0,00
Quantity of organic fertilizer (kg/ha)	0,01	0,00	0,01	**0,59**	0,00	0,04	0,08	0,13
Age of plantations (year)	0,04	0,01	0,00	**0,56**	0,05	0,01	0,01	0,02
Area of associated crops (ha)	0,20	0,09	0,11	0,00	**0,58**	0,01	0,01	0,00
Sowing method	0,20	0,00	0,01	0,38	0,09	**0,55**	0,03	0,03
Plantation density (plants/ha)	0,07	0,00	0,00	0,00	0,00	0,08	**0,55**	0,00
Cropping method	0,23	0,14	0,05	0,35	0,22	0,26	**0,70**	0,03
Tractor use (%yes)	0,00	0,00	0,00	0,01	0,02	0,09	0,00	**0,80**
*Valeurs propres*	*2,52*	*2,06*	*1,88*	*1,45*	*1,35*	*1,07*	*1,01*	*0,98*
*Pourcentage de variance (%)*	*14,87*	*12,14*	*11,09*	*8,57*	*7,95*	*6,30*	*5,99*	*5,80*
*Pourcentage de variance cumulée (%)*	*14,87*	*27,02*	*38,11*	*46,69*	*54,64*	*60,95*	*66,94*	*72,74*

Note: Only factors with an eigenvalue greater than 1 were considered.

CF: Chemical fertilization; IS: Irrigation system; LI: Labor intensity, OF: Organic fertilization; ECA: Extent of crop association; ST: Sowing technique; CD: Crop diversity; M: Mechanization.

The highest value of Calinski-Harabasz indicates the presence of three (3) types of plantain-based cropping systems, which is the “optimal k” number of clusters (Table 3).

**Table 3:** Number of system types and value of Calinski–Harabasz pseudo-F.

Number of types (k)	Calinski–Harabasz pseudo-F-index
**2**	49,00
**3**	**88,86**
**4**	78,97
5	85,92
**6**	86,93
**7**	87,89

**Fig. 2:**
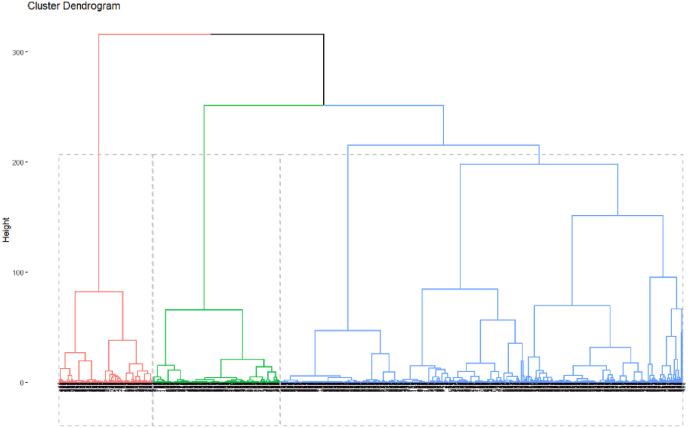
Dendrogram showing the three types of plantain-based cropping systems.

The visualization of the dendrogram confirmed that 3 “types” were obtained (Fig. 2).

The clustering solution was validated by the non-parametric robust statistical approach of bootstrap stability based on the Jaccard index (Table 4). It shows that the average stability values are greater than 0.85, indicating that all three types are stable and represent robust plantain-based cropping system types.

**Table 4:** Results from bootstrapping indicators.

Indicators	Type 1	Type 2	Type 3
**Count by type**	319	256	149
**Proportion (%)**	44,07	35,35	20,58
**Cluster stability**	0,90	0,89	0,86

Note: Bootstrapping replication size **is** 1000.

#### 3.1.2 Labeling and characterization of the types of plantain-based cropping systems identified

The identified cropping system types are labeled and described based on the sign of the two highest median values of the eight factors. Five of the eight factors highly influenced the types labeling (Table 5). The factors related to the ***“****irrigation system”, and* ***“****organic fertilization”* are the two dominant factors defining type 1. Farmers of this type are characterized by a non-irrigated plantain orchard, with a high use of organic fertilization. This type is therefore labeled *“Organic Dryland Cropping System”*. The factors *“chemical fertilization*” and *“*sowing technique*”* have the highest median values and dominate in the definition of type 2. This type is labeled " *Backyard Gardens and Traditional Cropping System*”. The use of*” chemical fertilization*” and *“irrigation system”* of farmers in the type 3 contrasts with other types and their respective cropping systems. This type is also characterized by the use of mechanizaion for land preparation. Type 3 is therefore labeled *“Intensive Cropping System."*

**Table 5:** Contribution of the seven factors to the median values of each type.

Factors	Type 1	Type 2	Type 3
Chemical fertilization and ecology	−0,28	−10,19	+20,04
Irrigation system	+0,24	+0,27	−10,74
Labor intensity	+0,06	+0,07	+0,37
Organic fertilization	−0,96	+0,25	+0,18
Extent of crop association	+0,06	−0,20	−0,57
Sowing technique	+0,18	−0,36	−0,14
Crop diversity	−0,16	+0,17	+0,00
Mechanization	−0,01	−0,10	+0,38

#### 3.1.3 Description of the types of plantain-based cropping systems

##### 3.1.3.1 Type 1: Organic Dryland Cropping System (n = 44.07%)

**Fig. 3:**
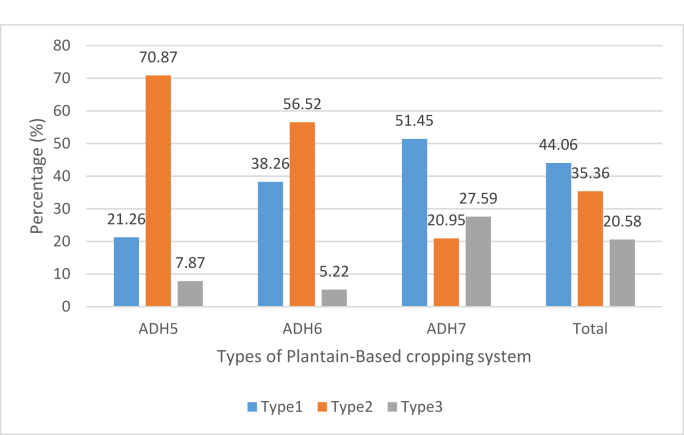
Distribution of plantain-based cropping system types by ADH.

The first type includes 44.07% of farmers' plots with an average age of plantation of 4.48 ± 3.34 years. This type is characterized by a high use of organic manure, i.e. 145.76 kg.ha-^1^. The dominant sowing method used is the row seedlings, which is observed at 90.60%. In terms of labor use, this type uses a low quantity of hired and total labor, respectively 4.55 ± 1.03 Man.day^−1^ and 13.06 ± 29.91 Man.day^−1^. The use of chemicals (urea and NPK) is similarly low, at 0.40 ± 0.12 kg ha^−1^ and 0.32 ± 0.09 kg.ha-1, respectively. Most plantations of this type (77%) have a density of less than 1200 plants per hectare (Table 6). The most important crop associated with plantain are maize (22.22%), dessert banana (21.64%), garden crops (tomatoes and chilli) (20.47) and oil palm (15.20%). Organic Dryland Cropping System is highly present in ADH7 (Fig. 3).

**Table 6:** Characterization of plantain-based cropping systems.

Variables		Type 1	Type 2	Type 3
Quantity of hired **labor** (Man.day^−1^)		4.55 ± 1.03^a^	2.90 ± 5.24^a^	20.10 ± 18.57^b^
Quantity of total **labor** (Man.day^−1^)		13.06 ± 29.91^a^	10.25 ± 8.65^a^	78.03 ± 5.45^b^
Quantity of urea (kg.ha^−1^)		0.40 ± 0.12^a^	0.07 ± 1.25^a^	20.37 ± 12.63^b^
Quantity of NPK (kg.ha^−1^)		0.32 ± 0.09^b^	0.00 ± 0.00^b^	21.67 ± 14.34^a^
Irrigated lowland (%yes)		0.00 (0.00)	0.00 (0.00)	60 (40.27)
Flat land (%yes)		240 (75.24)	244 (95.31)	17 (11.41)
Area of associated crops (ha)		0.34 ± 0.60^a^	0.07 ± 0.13^b^	0.26 ± 0.39^a^
Quantity of organic fertilizer (kg.ha^−1^)		145.76 ± 82.51^a^	36.94 ± 13.09^b^	7.02 ± 6.58^c^
Age of plantations (year)		4.48 ± 3.34^c^	7.02 ± 6.58^a^	5.79 ± 4.39^b^
Plantation density (plants/ha)	Low <1200 plants/ha (%)	248 (77.74)	239 (93.36)	116 (77,85)
	Average [1200–1500 plants/ha] (%)	71 (22.26)	17 (6.64)	33 (22,15)
Cultivation system	Relay cropping (%)	11 (3.45)	1 (0.39)	2 (1,34)
	Intercropping (%)	18 (5.64)	1 (0.39)	12 (8,05)
	Mixed cropping (%)	142 (44.51)	159 (62.11)	90 (60,40)
	Monocropping (%)	148 (46.39)	95 (37.11)	45 (30,20)
Plot irrigation (%yes)		1 (0.00)	0 (0.00)	117 (78.52)
Sowing method	Natural mat expansion (%)	21 (6.58)	122 (47.66)	30 (20,13)
	Row seedling (%)	289 (90.60)	0 (0.00)	89 (59,73)
	Staggered seedlings (%)	9 (2.82)	134 (52.34)	30 (20,13)
Tractor use (%yes)		6 (13.33)	7 (15.56)	32 (71.11)

Note: ANOVA test and Kruskall wallis test showed the characteristics common to each type. The means sharing a letter in the group label are not significantly different at the 5% level.

##### 3.1.3.2 Type 2: Backyard Gardens and Traditional Cropping System (n = 35.35%)

This type comprises 35.35% of farmers' plots, with an average age of plantation of 7.02 ± 6.58 years. This type is characterized by the absence of chemical fertilizers (NPK and urea) on these plots. Similarly, labor use is the lowest, at 2.90 ± 5.24 Man.day^−1^ and 10.25 ± 8.65 Man.day^−1^, respectively. The majority of these plots are found on flat land (95.31%) and characterized by a low area allocated to associated crops (0.07 ± 0.13 ha). Still, the proportion of crop association iss high (62.11%). Among the crop associated, dessert banana stands as the most important (44.1%). Natural mat expansion (52%) and staggered seedlings (47%) are the most common sowing methods used in this type. Backyard Gardens and Traditional Cropping System are mostly present in ADH5 and 6 (Fig. 3).

##### 3.1.3.3 Type 3: Intensive Cropping System (n = 20.58%)

Type 3 mobilizes (20.58%) of farmers' plots. As indicated by its label, this system is characterized by an intensive use of total labor (78.028 ± 5.45) and hired labor (20.10 ± 18.57). It is also characterized by higher fertilizers (urea and NPK) use than other types. The average amount of urea and NPK used are 20.37 ± 12.63 kg.ha-1 and 21.67 ± 14.34 kg.ha-1, respectively. Plots of this type are partly located on irrigated lowland (40.27%) and 78.52% of plots benefit from an irrigation system. Farmers belonging to this type mainly use mixed cropping (60.40%) and monocropping (30.20%) on their plots. Maize is the most important crop associated with plantain for this type (34,62%). Cowpea is also associated to plantain but on only 10.57% of plots. Tractor use is mostly observed in this type at 71.11%.

### 3.2 Constraints to plantain production

The results of Kendall's rank correlation revealed Kendall's W values greater than 0.5 and highly significant overall (Table 7). These values reflect a good level of consensus among plantain farmers in the constraints ranking. Moreover, by referring to the Chi-square distribution table, all the $x^{2}$ values obtained are higher than the critical values of $x^{2}$, which are 26.30 and 32.00 at 5% and 1% significance, respectively, with a degree of freedom df = 16. Thus, it can be concluded with a considerable confidence that the consensus among the respondents is higher than it would have been by chance if their ranking had been random or independent. The results further shows that drought, wind, and flood are the most important constraints, and animal damage is the least important constraint in general for the overall sample regardless the type. The top three factors identified are climatic hazards resulting from climate change which induce higher temperatures, irregularity in rainfall distribution, as well as a drop in rainfall. The content analysis carried out confirm this tendency. As matter of fact, it reveals that climatic hazards are among the main factors limiting plantain production. Various causes and consequences are highlighted in the verbatims. According to farmers, water is an essential factor limiting plantain production. *‘‘Plantain means water. It is the beginning and the end of the production’’ (Participant Discussion Group (PDG), Tori-Bossito, 2021)*. *‘‘Water is the only thing that limits us and has always been a problem for us on the 4 ha we have rented’’ (PDG, Akpro-Missérété, 2021)*. Some farmers believe that community meteorologists (commonly called rainmakers) prevent rainfall for funerals, fishing, or road construction purpose, causing drought. Others perceive this constraint as a simple climate change consequence. The lack of water creates water stress on the plant that considerably reduces the bunch's weight. Also, under windy conditions, plants fall over, causing production losses. *‘‘The plants lack water. And man is the cause of the problem. when there is a funeral, the one who holds it consults the rainmaker, who help him to prevent the rain from falling and blow wind into it. When it doesn't find water, it withers and breaks at the slightest gust of wind’*’ *(PDG, Zè, 2021)*.

**Table 7:** Prioritization of constraints related to plantain production.

	Type1		Type2		Type3		Total	
	AV.R.	Rank	AV.R.	Rank	AV.R.	Rank	AV.R.	Rank
Drought	2.09	1	1.00	1	1.00	1	1.00	1
Wind	2.00	2	3.00	3	2.00	2	2.00	2
Flood	4.00	3	5.09	5	4.00	3	4.00	3
Low farm-gate price	4.00	4	3.00	2	4.00	4	4.09	4
Difficulties in accessing the market	6.00	5	5.00	4	5.00	5	6.00	5
Lack of credit facility	6.00	6	6.10	7	6.00	6	6.00	6
Theft	6.00	7	6.00	6	6.00	7	6.00	7
Pest and diseases	7.00	8	7.00	8	7.00	8	7.00	8
Decline in soil fertility	8.00	10	8.00	9	8.00	9	8.00	9
No standard measurement unit	7.00	9	8.00	10	8.00	10	8.09	10
Access to land	10.00	11	10.00	11	10.00	11	10.00	11
Lack of technical support	11.00	12	11.00	12	11.00	12	11.00	12
High input prices	12.00	13	12.00	13	12.00	13	12.00	13
High labor cost	13.00	14	13.00	14	13.00	14	13.00	14
Unavailability of clean planting materials	14.00	15	14.00	15	14.00	15	14.00	15
Poor transportation system	15.00	16	15.00	16	15.00	16	15.00	16
Animal damage	16.00	17	16.00	17	16.00	17	16.00	17
Kendall's W^a^	0.84***		0.85***		0.84***		0.84***	
$x^{2}$	4203.00		3460.01		2070.00		9720.76	

Note: AV.R. means “average rank”, ***p < 0.01.

A deep analysis of Table 7 reveals slight differences in constraints ranking, most specifically for type 2. Drought, low farm-gate price and wind are the major constraints agreed by farmers in that type. Difficulties in accessing market is also an important constraint raised by farmers. Thus, beside climate hazards, marketing constraints appear to be of utmost importance. Different reasons are mentioned by farmers. First, traders make loans to farmers, putting them in the position of price takers. Second, the poor transportation system negatively influences prices. The attempts of farmers to get their plantains to market are in vain because of the barriers to entry. *‘‘We avoid selling our products at the market because it is complicated. Our market is so organized that when you got in, traders spot you. They wait for those who buy up to bring it to Cotonou. And you, farmer, with your products, cannot be patient. You have to sell to them. And they use this opportunity to lower the price and unsettle you so that you think twice before entering the market again’’ (PDG, Tori-Bossito, 2021)*. The lack of knowledge in planning harvests in times of scarcity, the inexistence of a price regulation institution as well as lack of a standard unit of measurement for bunches are other causes of the low price experienced by farmers. *‘If we could create a market where people will come and buy plantain* *per* *kilogram, it would be good. It would be a group sale. And we would also like to have contracts with customers’’ (PDG, Athieme, 2021)*. The non-existence of more profitable markets able to guarantee good prices lead farmers to sell their products at low prices to reduce the risk of product perishability. Besides, farmers are subject to price volatility. *‘‘Plantain prices have dropped*. *What happens is that when you have your plantain, you cannot sell it at the price you want ‘’ (PDG, Akpro-Missérété, 2021).* This market constraints negatively affect farmers' income and, consequently, their profits.

Financing is also a considerable constraint to plantain production. Farmers argue that plantain plantations require a high level of capital for the establishment, which is difficult to find. *‘‘First, if you produce and you don't find the right market to sell to, and if you don't find financing and* support*, you can't go far. That's our challenge ‘’ (PDG, Athieme, 2021).* Similarly, regular maintenance requires working capital that comes either from other activities carried out by farmers or from informal loans (tontines, relations, traders) or from financing institutions where access to credit is difficult. *‘‘We do not have access to credit, the access conditions are complex and our files are constantly rejected’’ (PDG, Akpro-Missérété, 2021).* As a result, maintenance operations are postponed, causing resistance between plants and weeds. This situation negatively impacts plantain yields.

## 4 Discussion

The typology of plantain-based cropping systems identified three types of cropping systems in the study area: “Organic Dryland Cropping System”; “Backyard Gardens and Traditional Cropping System”; “Intensive Cropping System”. These types depict the map of plantain evolution in the country. As highlighted in Lokossou and Achigan (2000) plantain and banana were considered, in the past decades, as a secondary crop and was neglected to the extent that there were no statistics on the crop as it was not included in the country strategic agricultural policy document (Lokossou & Achigan, 2000). The features of the type 2 match the agricultural practices and the way banana and plantain were produced back then. The most striking feature that brings type 2 closer to traditional and backyard gardens cropping systems, is the sowing method. Farmers use either natural mat expansion or staggered seedlings. Because of the marginal place held by the crop back then, low care was given to plantain orchard translating into low labor use, low input use. Consequently, some activities like orchard cleaning as well as the planting remain hardly done. The principle of natural mat expansion is to carry out the planting once. The orchard size will then be increasing through the plantlets emerging from the mother plant, forming mats. Moreover, the staggered seedling coupled with the low area allocated to crop association and the high propension of mixed cropping signpost a traditional way of growing plantain. However, the advantage of this sytem is that fields are generally near to huts or houses, implying that the plants usually benefit from bath or domestic used water and waste produced by human and trees around. Lokossou et al. (2012b) also identified this type of plantain-based cropping systems in Allada's high-land in Benin. Besides, traditional cropping systems have been gradually shifting to modern plantain production in the production areas. The results showed to types of modern plantain production: the organic cropping system and the intensive cropping system, one at the opposite of the other. The first is not assimilated in this study to the certified organic production explored in Dassou et al. (2021), which is a strict process with practices inherent to the organic certification. The intensive cropping system is characterized by a high input use, the use of irrigation and agricultural equipment. This type of cropping system appears to be strongly market-oriented and applied by profit-seeking farmers who are well endowed. This is an indication that this type is composed of wealthier farmers and entrepreneurs. These results are in some points similar to those of Ahouangninou et al. (2021) who found three groups of banana farms in the district of Houeyogbé. These three classes include: backyard crop gardens, inter-cropping farms with average size and income and equipped large-size banana farms.

Considering inter-segment variations in our study, in two out of the three types of plantain-based cropping systems crop association is mainly used. This is due to the long production cycle of plantains (12–18 months) and the land use maximization objective for food security and additional income. The same reasons were raised by Lokossou et al. (2012b) and Lokossou and Achigan (2000) in the Allada's highland and in the departments of Mono, Ouémé, and southern Zou (ADH 7 and 5). Crop association also allows farmers to maintain the orchard. In fact, according to farmers, by sowing maize, legumes, or vegetables between the plants, plantains benefit not only from the field's maintenance and the lag effects of the inputs but also, from the decomposition of these plants after harvesting, thus playing the role of manure. This back up the features of type 2 as a low-care cropping system. As actual fact, dessert banana is the most important crop associated with plantain for type 2. As plantain and banana are similar species in terms of some physical characteristics, farmers are reluctant to apply care, that would have benefited plantain orchard if associated with cereals, legumes or garden crops. These results are consistent with those of Faïnou et al. (2018). Basically, during the plantain production cycle, different crops can be grown on the plot depending on the farmer's logic. Indeed, some farmers prioritize maize at the beginning of plantain cycle and cowpea at its end to avoid competition with the crop. According to this logic, maize does not compete with the plantain at the beginning of the cycle and can normally develop to the phase of bunch emergence. The association of plantain and legumes such as cowpea improve soil fertility and does not compete with plantain. On contrary, the association plantain-oil palm does not benefit plantains. Plots with this type of association are often in the process of restructuring, either by replacing oil palm with plantain or the opposite. Experiments conducted on crop associations with plantain by Lokossou et al. (2012b) showed that palm oil strongly compete with plantain for nutrients. However, rotation of the plantain-corn in the first rainy season with plantain-cowpea in the second rainy season has the best agronomic performance and contribute to the improvement of soil nutrient status (Lokossou et al., 2012b). Besides, fertilizers and pesticides applied to intercrops and the capacity of certain intercrops such as legumes to capture nitrogen present in the air to improve soil fertility made a favorable contribution to plantain growth (Dé et al., 2019).

Water is the basic input needed to establish a plantain orchard (Houssou et al., 2013; Lokossou et al., 2012b). When plantains suffer from a water deficit, the use of fertilizers or soil fertility does not prevent the production of thin bunches. Drought or sudden interruption of rainfall is therefore a limiting factor in plantain production (Akode, 2015). This explains the high level of consensus among farmers regarding the ranking of this factor as the most important one limiting their production. In addition, the amount of water supplied is also a factor that can decrease the productivity of the plantation. When more water is supplied than is necessary, it can lead to a reduction in yields (Houssou et al., 2013). Consequently, flood, is also an important limiting factor, most specifically in ADH7 (Adjohoun, Athieme, Grand-Popo). In addition to these factors, plantain is a very sensitive plant to the wind, causing important damage to the plant (fall of the pseudo-stem) as showed by Ngo-Samnick (2011). This helps explain the importance of this hazard for all types.

The marketing of plantain is also a challenge for farmers. The low prices observed, the lack of a standard unit of measurement and, the difficult access to a more profitable market have been reported as important across all types. This situation makes transactions more difficult, with actors in a power position dictating prices to farmers who are « price takers» (Dzomeku et al., 2011). These market factors were also reported by Ayanwale et al. (2016) and Schill et al. (2000). Establishing a plantain orchard is a fairly high investment. Farms that do not have enough financial means to support these expenses express a need for financing. This is an important constraint, as liquidity allows the farmer to hire labor for field maintenance, apart from the first year of heavy investment. This is also a very important constraint in some West African countries such as Ghana and Nigeria (Ayanwale et al., 2018; Tomekpe et al., 2011). The lack of financing can lead to the non-adoption of innovations, the low level of risk management strategies' use and increase farmers’ vulnerability to climatic hazards (Ogouvide, 2020; Ojo and Ayanwale, 2019, Sossou, 2015).

## 5 Conclusion

Plantain cropping practices have evolved and diversified over the time, leading to a heterogeneity in cropping systems. The latter is necessary to be accounted for when devising effective policy interventions. In this study, we use a multivariate analysis to construct a typology, and by this way, capturing the diversity of plantain-based cropping systems in Benin. This approach has been combined with the Calinski-Harabasz pseudo-F statistic, the non-parametric bootstrapping, and the ANOVA to yield an optimal and robust number of clusters. The results revealed three “types” of plantain-based cropping systems: “Organic Dryland Cropping System”; “Backyard Gardens and Traditional Cropping System”; “Intensive Cropping System”. They differed from each other by the seedling method, cropping system, water management, labor use intensity, and input use intensity. Only one type (type 3) is characterized by an additional water supply, thereby not relying on the climate. It is also characterized by a high use of fertilizers and labor. This type is dominant in the ADH 7, indicating that ADH7 is an important area of plantain production in Benin. Furthermore, some constraints such as drought, wind, flood are the top three for the types and in general except type 2. Still, drought stands out as the first constraint and low farm gate price as the second constraint which farmers agreed on for that type. It implies that regardless the diversity, plantain farms are exposed to climate and price risk. Nevertheless, some factors such as, lack of measurement standards, financing constraints, pests and diseases, and difficult market access are important and vary from one type to another. Consequently, interventions need to be type-specific and tailored to achieve expected efficiency and impact. More actions are needed to reduce the effect of drought, wind and flood for types 1 and 3. Interventions toward price and market are of utmost important for the type2. Although our approach allows for a robust identification of plantain-based cropping systems in Benin, it has some limitations that are important to address in future studies. First, the approach is based on static data and does not account for a probable dynamic feature of farms and farmers. Second, it does not account for farmers’ perception of differences or similarities in the farms which might contribute to reflecting more on the realities of the environment. Third, the non-parametric bootstrap used is likely to overestimate the stability of the clusters. Despite these drawbacks, our study contributes to provide more information on the diversity of plantain-based copping systems existing in Benin using a statistical approach. This diversity has been accounted for in the constraints analysis, calling for tailored future interventions. It thereby contributes to fill this gap in the literature.

## CRediT authorship contribution statement

**Adikath Abiola:** Conceptualization, Data curation, Formal analysis, Investigation, Methodology, Project administration, Visualization, Writing – original draft, Writing – review & editing. **Ygué P. Adégbola:** Conceptualization, Methodology, Project administration, Supervision, Validation, Writing – review & editing. **Martine Zandjanakou-Tachin:** Funding acquisition, Project administration, Validation, Writing – review & editing. **Géraud F. Crinot:** Data curation, Formal analysis, Writing – original draft, Writing – review & editing. **Gauthier Biaou:** Project administration, Supervision, Validation, Writing – review & editing.

## Declaration of competing interest

The authors declare that they have no known competing financial interests or personal relationships that could have appeared to influence the work reported in this paper.
